# An ethical framework for human genomic enhancement in China

**DOI:** 10.3389/fgene.2025.1600829

**Published:** 2025-05-14

**Authors:** Fanhong Chen, Hongjiao Zhang

**Affiliations:** Law School Central South University, Changsha, Hunan, China

**Keywords:** human genomic enhancement, CRISPR-Cas9, ethics, gene editing technology, China

## Abstract

Human genomic enhancement, or HGE, which improves human traits by introducing genetic and epigenetic changes, has garnered a lot of attention in light of the astounding advancement in genome editing techniques, such as CRISPR-Cas9, in recent years. This study combines doctrinal and empirical analysis methods to examine bioethical issues of HGE in China. The literature currently in publication on these issues indicates that the majority of Chinese academics and the general public are enthusiastic about the present and potential future applications of genome editing techniques within the parameters of appropriate ethical guidelines; regrettably, no workable ethical governance framework has been put forth so far. Considering this, this study offers a more comprehensive discussion of ethical policy on HGE and develops a robust ethical framework that addresses three related issues about HGE: (1) approaching the precautionary principle as an overarching benchmark; (2) producing a multi-stakeholder collaborative governance model to promote stakeholder engagement and dialogue; and (3) establishing regional ethics review centers to have an independent review process. This is done in an effort to address ethical concerns and further inform policymaking on HGE in China.

## Introduction

Human genomic enhancement (HGE) is an artificial process that involves “the introduction of changes into the genome to modify and improve nonpathological human traits” (Yasemin and Philip, 2022). While HGE holds out the promise of improved human capacities, it also raises ethical concerns because of the threat it poses to “our shared human nature,” “human dignity” and “social justice” (Araujo, 2017). As a response, traditionally, the discussions and debates over the ethical issues of HGE uses a typological approach by defining the differences between somatic and germ line/heritable gene editing, as well as between gene editing for enhancement and therapy (Howard et al., 2017). This approach implies that a genetic intervention can only be carried out if it is done so for therapeutic, diagnostic, or preventive purposes and if it does not alter the genome of any future generations.

This attitude has, however, recently come under scrutiny in China due to the widely recognized and controversial blurring of the lines separating therapy from enhancement, the international development of ethical guidelines on HGE, an open attitude of Chinese academia and the public toward HGE, and the urgent need for the application of genome editing techniques in certain industries, such as aging delay and silver economy development in China. As a result, a thorough discussion over new ethical guidelines is required.

## Human enhancement, genome editing technology, and human genomic enhancement

One of humanity’s greatest pursuits is to enhance human beings’ performance beyond what is considered the normal or typical (Anomaly and Johnson, 2024). For thousands of years, leaders in most, if not all, countries have been searching for methods to improve their physical and longevity wellbeing. Throughout the history of the 1930s and 1960s, various supporting policies relating to “population control, social hygiene, public health concerns, and sexual education” have been implemented within nations to make people “better” or “higher” (Güvercin and Arda, 2008). Whether successful or not, it is clear that these approaches represent a widely held pursuit—that of human enhancement. In this sense, human enhancement points to a wide range of interventions and technological advancements, such as machines, that aim to improve human performance by modifying human traits and optimizing human capabilities (NHGRI, 2018).

In the biomedical field, genome editing technology, which has advanced rapidly to alter, remove, or add particular genes in adult subjects or the germline, implies its potential application for enhancement purpose (Blackburn and Rowley, 2004) (Park and Bae, 2024). Initially, when genetic engineering first emerged, scientists could only modify genes outside of cells. In the early 1970s, changes were made inside bacteria using viruses that added genes. With the advent of Zinc-finger nucleases (ZFNs) and the development of transcription activator-like effector nucleases (TALENs) technology in the 2000s, gene targeting gradually became more precise but was still quite cumbersome and expensive. In 2012, the CRISPR (Clustered Regularly Interspaced Short Palindromic Repeats)-Cas9 system stood out as the most powerful genome editing tool, as it allows geneticists and medical researchers to target any part of the genome in the fastest, cheapest, and most accurate manner (Jinek et al., 2012). These attributes make CRISPR-Cas9 the best option for somatic or germline gene editing to improve human performance. In this study, therefore, we refer to the application of gene editing technology for the purpose of human enhancement as human genomic enhancement (HGE).

The concept of HGE is often defined in opposition to therapy or medical treatment that intends to restore the condition of an individual from existing injuries or heal undesired diseases that are already present (Norman, 2000; David, 2000). In theory, the line between the two concepts and the according standards can be sharply defined, as enhancement targets interventions to “add capabilities” beyond the normal range while therapy focuses on means to “recover capacities” back to the normal level (Du, 2018). In practice, however, they may merge into each other for two reasons. First, the field of medicine has gradually expanded. In addition to traditional therapy, interventions like “preventive medicine, palliative care, sports medicine, contraceptive procedures, and fertility procedures” have all fallen within the scope of medicine (Raposo, 2022). Second, the concept of “normal” varies within different social perceptions. For example, the average height of people in developed countries has increased by around four inches over the past 150 years. Therefore, the line that separates enhancement from therapy is unclear, poorly understood, and possibly unstable.

Because of the conceptual demarcation between therapy and enhancement, certain safe and effective researches for therapy might readily be applied for enhancement that is carried out to a degree beyond normal health. In 2015, in an attempt to find a cure for beta-thalassemia, a genetic blood disorder, some Chinese scientists first released a research that applies CRISPR-Cas9 in an experiment utilizing 86 non-viable human embryos (Liang et al., 2015). Only 3 years later, a Chinese scientist, He Jiankui, created the first human genetically edited twin babies, Lulu and Nana. The babies’ genes were modified using CRISPR-Cas9, and as a result, they increased resistance to HIV infection (Baylis, 2019). It can be seen that parents could be tempted to improve their babies’ traits in accordance with their preference through genome editing in IVF. We argue that compared with the genomic intervention for the existing disease (e.g., beta-thalassemia), acquiring higher protection against HIV through genome editing is very much like enhancement rather than mere therapy. Therefore, CRISPR-Cas9, which holds tremendous promise for genome editing for the purpose of therapy, could conceivably pave the way for enhancement (Araujo, 2017).

Given the limitations of the therapy/enhancement dichotomy, some scholars have proposed a welfare-based approach, which defines HGE as “any change in the biology or psychology of a person which increases the chances of leading a good life in the relevant set of circumstances.” (Savulescu et al., 2011) According to this approach, gene editing interventions are only deemed enhancement if they really promote human wellbeing. This approach presents a main concern that the concept of welfare is too vague, making it difficult to apply. This paper argues that in alignment with Chinese culture, HGE shall take into account the interests of both individuals and society. Accordingly, there may exist four scenarios: (1) both personal welfare and social welfare are improved; (2) individual welfare is boosted while social welfare is compromised; (3) social welfare is enhanced while individual welfare is compromised; (4) neither individual nor social welfare is improved. Consensus could be comparatively easy to attain for the first and fourth scenarios. The second and third scenarios, however, may provide varying results based on the customs and regulatory frameworks of distinct ethnic groups and nations.

## Development of ethical guidelines on HGE all over the world

The enormous capacity of technical breakthroughs to intervene in the human genome is accompanied by serious ethical concerns. First, Francis Fukuyama argues that “The original purpose of medicine is, after all, to heal the sick, not to turn healthy people into gods” (Fukuyama, 2002). Second, a UNESCO-affiliated international bioethics committee states in the “Report of the International Bioethics Committee (IBC) on Updating Its Reflection on the Human Genome and Human Rights” that HGE poses a threat to human dignity (UNESCO: United Nations, 2015). Third, HGE may represent a challenge to distributive justice, because individuals who achieve enhancements could gain a certain level of advantages over others. This social inequality will be intensified if only the monetarily privileged gain access to HGE in the market (Anomaly, 2020). Fourth, HGE presents a threat to human autonomy, as HGE may not only jeopardize offspring’s self-determination but also potentially contradict their right to an open future (Feinberg, 1980). Lastly, HGE may adversely impact relational ethics, which underscores individuals’ need to cultivate robust and meaningful connections with others (Pollard, 2015). This is because HGE would erode the unconditional love and nurturing gratitude parents hold for their children. In this context, to fully understand the variation of organisational or expert body statements on the ethics of HGE, we reviewed and analyzed representative ethical guidelines worldwide from 2015 to 2024. Specifically, we historically examined what stances these guidelines take on HGE and if and how these stances impacted HGE research.

The first stage of ethical standards centers on differentiating between somatic and germline gene enhancement as well as between gene editing for the purpose of enhancement and therapy. It began immediately after CRISPR-Cas9 was initially used in human embryos that were not viable. In 2015, when the CRISPR-Cas9 technique was still very much in the development phase, a statement titled “The Opportunities and Limits of Genome Editing,” jointly released by four scientific agencies in Germany, cautiously emphasized the important potential of scientific research on genome editing, with particular regard to the distinction between gene therapy of somatic cells that will not affect the human germline and inheritable genetic changes (National Academy of Sciences Leopoldina, 2015). The next year, the Opinion Group of the Bioethics and Law Observatory of the University of Barcelona launched a Declaration on Bioethics and Gene Editing in Humans, stating that any clinical application of germline genome editing should not be used for human enhancement. The EU has, predictably, adopted the same stance. In 2017, Federation of European Academies of Medicine expressed its concerns over the creation of individuals with enhanced human capabilities and explicitly opposed genome editing, including somatic genome editing, for non-medical interventions (A position paper of FEAM - the Federation of European Academies of Medicine, 2017).

The ethical standards in the second stage redefined the concept of HGE and disregarded the typological approach in the first stage. The Nuffield Council of Bioethics published “Genome Editing and Human Reproduction: Social and Ethical Issues,” a representative report on genome editing in relation to reproduction, in 2018. To a certain extent, as we discussed above, heritable gene editing as a reproductive technology used by individuals might fall out of the scope of therapy because of the absence of an existing disease in current individuals. However, the report has changed course and acknowledged the challenges to differentiate between enhancement and therapy. Probably because of such difficulty, it creates an alternative strategy that centered on social norms and individual welfare, rather than concrete practices and applications, to assess whether heritable gene editing is morally permissible. In light of this, scholars argued that any application, including genome editing for human enhancement, could be morally acceptable as long as it advances individual wellbeing and social solidarity (Gyngell et al., 2019).

In the final stage, the SIENNA project more recently offered a stand-alone ethical analysis for human enhancement technologies, thoroughly examining HGE as a case study. While it may not be seen as taking a pro-enhancement viewpoint, the analyzing framework is nonetheless important to pay attention to. For instance, to deal with technological risks and side effects, an institutionalized independent ethical review process is needed to assess each potential application of gene editing technology on a case-by-case basis. The possible privacy infractions call for new protocols and security measures that are specific to protect genomic information. Another well acknowledged ethical concern is eugenics and the resulting unfairness. According to the report, it is imperative to avoid genome editing “on a large-scale societal level with the sole purpose of promoting certain features that aim at altering human nature beyond restoring health.” It is also important to note in the report that these suggestions might produce nuanced outcomes, taking into account local traditions and values in different countries.

## Current regulatory framework of HGE in China

Ethical concern over gene editing technology has spread significantly in the wake of the He Jiankui event (Sand et al., 2019). Accordingly, China’s legislation pertaining to genome editing has undergone substantial improvement. The Civil Code went into effect in January 2021, and it has new sections on science and technology ethics that govern medical and scientific research activities related to human genes and human embryos. The Criminal Law’s Amendment (XI) in March 2021 clarifies criminal criteria and adds provisions on criminal responsibility for acts such as cloning human embryos and violating ethical norms of gene editing. In April 2021, China passed the Biosafety Law, which lays out the ethical guidelines for the gathering, preservation, and use of human genetic resources. Based on this, the Regulations on the Management of Human Genetic Resources (the *Regulation*) stipulate that appropriate actions (including the human embryo gene editing as a laboratory research rather than clinical research or medical practice) (Chen et al., 2022)pertaining to human heritage resources should be approved by an expert review committee made up of experts from various disciplines, after receiving informed consent from all relevant parties. Later in January 2022, the relevant ethical standards are further clarified by the Science and Technology Progress Law. In summary, research on genome editing is allowed, but it must adhere to some fundamental legal standards that protect human heritage resources.

The above fundamental standards played a role in shaping the “Opinions on Strengthening the Ethical Governance of Science and Technology” (*the Opinions*) released in March 2022, by the State Council and the General Offices of the Communist Party of China Central Committee. This document, as a top-level design, prioritizes ethical values and establishes ethical requirements as a precondition for the use of genetic technology. To implement the *Opinions*, in October, the Ministry of Science and Technology, together with ten departments including the Ministry of Education, the Ministry of Industry and Information Technology, and the National Health Commission, jointly issued the Measures for Ethical Review of Science and Technology (Trial Implementation) (*the Measures*), which put forward concrete requirements on the basic procedures, standards, and conditions of ethical review of science and technology. First, it proposed that the promotion of innovation should be unified with the prevention of risks. Second, the entity, including but not limited to universities, scientific research institutions, medical and health institutions, enterprises, is responsible for carrying out the ethical review of science and technology. Third, it establishes ethics review committees and the registration system of high-risk scientific and technological activities in ethics of science and technology. Fourth, to ensure effective implementation, the *Opinions* lays forth the principles of “promoting human wellbeing, respecting the right to life, preserving justice and fairness, reasonably controlling risks, and maintaining openness and transparency.” In view of these requirements, it seems that under close ethical examination, human genome editing for the purpose of enhancement could be approved.

## Increasing debates on ethical issues of HGE in China

In recent years, there has been a lot of discussion about the ethical issues surrounding HGE in both academic and public settings. In order to present the complete picture of the controversy, we conducted a search in major social science journals, newspapers, and social media platforms for publications that contained the keywords of ethics and gene enhancement. The search results were then quantitively and qualitatively analyzed.

To explore academic debates on HGE in China, we conducted a search of the China Academic Journal Network Publishing Database (CNKI) on 1 April 2024. We accept that the articles in other databases that cover relevant materials may be overlooked if the analysis is restricted to academic publications from CNKI. While this is not ideal, we do think that most scholarly work that is published in the main journals are included in CNKI in China. We searched for and collected all articles that contained the keywords “ethics” and “gene enhancement” dated between 1 January 2003 and 1 April 2024. The period of analysis, 2003–2024, was selected because we wished to examine academic opinions over a significant length of time. The search returned 105 articles, of which 29 were identified as false positives, leaving us with 76 articles. And by false positive, we mean articles that merely introduced literature about HGE in other countries, that were related to ethical issues of gene research in a broad sense, and that analyzed human enhancement based on other technologies such as AI.

From a qualitative perspective, there are two camps among Chinese academics regarding HGE: proponents and opponents. It seems that neither academics in favor of nor against HGE can totally persuade the other. Specifically, proponents of HGE in Chinese academic circle argue that, given the intrinsic malevolence of human nature (Bostrom, 2005), HGE offers a viable substitute for compassion and social justice, positing that through gene editing technology and future technological advancements, humanity can ethically modify natural human characteristics in alignment with humanitarian values and individual aspirations, thus perpetually refining human nature (Jiang, 2004). They also contend that, far from causing inequality, HGE will furnish individuals with superior physical, psychological, and intellectual foundations to pursue personal dignity in light of the Confucian traditions in China (Li, 2019). By contrast, beginning with the integrity of human nature, opponents of HGE, especially HGE for nonmedical purpose, contend that genetic enhancement violates human dignity, would lead to an identity crisis for individuals and following generations, has a negative influence on social justice and fairness, and is an infringement on nature’s order (Chen, 2021).

From a quantitative perspective, the overall conclusion from the 76 articles is that human enhancement is widely divided by Chinese academics between medical and nonmedical purposes, with only 22.37 percent of the discussion objecting to nonmedical HGE. In other words, the majority of Chinese scholars (as each scholar wrote only one article in the dataset) supported HGE provided that it was properly regulated and morally supervised. Regrettably, however, in the literature review, no workable ethical governance framework has been put forth so far.

In addition to academic debates, HGE is currently the subject of policy and media discussions. It is widely acknowledged that genome editing could, in certain cases, increase human lifetime beyond existing natural limits. Such interventions into human nature go beyond simply restoring the normal functioning of human physiology and can therefore be viewed as HGE. In January 2024, China released its first silver economic policy document, Opinions on Developing a Silver Economy to Improve the Wellbeing of the Elderly. Expanding research on human aging models, skin aging mechanisms, human hair health, and the development and implementation of gene technology and regenerative medicine in the field of anti-aging are some of the initiatives that have garnered a lot of attention. Major media outlets are vying with each other to report on the significant contribution that gene editing technology will make to the growth of the silver economy. In this respect, we believe that the beneficial effects of gene editing technology in postponing aging will come true in the future.

Furthermore, HGE has sparked a great deal of public debate, particularly in the wake of the He Jiankui event. A search on Google for the terms “gene editing babies” yields a total of 384,000 unique hits. Additionally, in 2019, a number of Chinese academics conducted an empirical research on the public’s opinions on gene therapy and enhancement as well as the factors that influence the degree of acceptability (Zhang et al., 2023). In this research, 761 valid questionaires were collected. The data analysis suggested that public opinions can be divided into four categories. The largest percentage—nearly half of the subjects underwent both gene therapy and gene enhancement concurrently; the second, which makes up around 30% of the sample, is the mindset that only supports therapy and not enhancement; over 20% of the people do not support either technology; the lowest percentage (1.9%) advocated just enhancement rather than therapy. Therefore, it can be said that there is not an absolute objection from the Chinese people to HGE.

## The proposed ethical framework

After examining the ethical discussions surrounding HGE in China, we then develop a robust ethical framework that can inform policymaking on HGE, based on the regulatory culture in China. Following the international development of the ethical guidelines, this framework does not develop for each enhanced human function or divergent purpose, but addresses three related issues: (1) approaching the precautionary principle as an overarching benchmark; (2) producing a multi-stakeholder collaborative governance model to promote stakeholder engagement and dialogue; and (3) establishing regional ethics review centers to have an independent review process.

### China’s unique regulatory culture

Confucian ethics significantly influence the distinct regulatory cultures in China. First, Confucianism requires that personal autonomy be subordinate to the sustainable development of the family. Although some family collective decisions may be potentially detrimental to individual interests, individuals are obliged to adhere to these decisions based on the principle of filial piety. (Thomas Edison State University and Alverson, 2025) Second, the traditional Confucian notion of dignity encompasses universal dignity, signifying that all individuals inherently contain moral potential, and personal dignity, indicating that individuals can attain dignity to differing extents by their acts. This should be taken into account in the ethical evaluation of HGE. Thirdly, it is worth noting that the relationship between ancestors and descendants, as well as between parents and children, lacks equality; rather, its core is fundamentally rooted in obedience and authority. In this regard, if HGE can rectify deficiencies in genetic inheritance to ensure the perpetuation of family lineage, parental actions regarding HGE may be endorsed, and offspring should acquiesce (Zhu, 2020).

Influenced by Confucian ethics, China’s regulatory culture displays two distinct characteristics. The first one is the government-driven regulation model. Government-driven regulation denotes the Chinese government, as opposed to research institutions, researchers, technological firms, and other stakeholders, playing the paramount role in the advancement of science and technology in China (Bradford, 2023). Considering that the Chinese government prioritizes social stability and harmonization as important policies, the advancement of HGE should be contingent upon these factors. Moreover, in contrast to the individualistic culture that emphasizes personal achievements and interests, a collectivist culture prioritizes collective interests over individual interests, necessitating that individual interests concede when they conflict with collective interests. This can be inferred from China’s slogan emphasizing the concentration of efforts to achieve significant objectives in various fields.

### Toward the precautionary principle

As previously mentioned, the most recent *Opinions* places a high priority on ethical values and, as a result, creates ethical principles for the application of genetic technology. These principles are key to improve the objectives and means pertaining to HGE. However, when implementing these principles in practice, we suggest the precautionary principle as an overarching benchmark in the ethical evaluation of HGE, which involves risks and uncertainties regarding potential harm and its magnitude. Although the precautionary principle lacks a standardized definition, it is widely accepted that this principle prioritizes risk anticipation and harm prevention (Koplin et al., 2019). Under China’s regulatory culture, HGE encompasses both physical health and societal stability, with associated dangers comprising individual and societal risks. In contrast to conventional risk-based decision-making approaches, the decision-making framework informed by the precautionary principle possesses the following three characteristics.

First, the precautionary principle does not emphasize the minimal degree of safety and the maximal degree of danger, both of which are considerably uncertain. Instead, it emphasizes problem-solving strategies, such as risk mitigation or alternative solutions, while ensuring essential benefits. This is because the effects of reducing risks, such as off‐target mutations, can become progressively evident through technological advancement. Following this substantive requirement, risks associated with HGE in a specific context should assess against the extent to which risks can be mitigated while preserving essential benefits, the availability of alternative activities, the necessity of HGE, and its ethical concerns in that particular context.

Second, the precautionary principle is preventive, and thus requires that the burden of proof for any activity involving technology that could endanger the health or public safety must be on the person advocating for the use of those technologies rather than the public (The Global Development Research Centre, 1998). Following this procedural requirement, the proponents of HGE should bear the burden of proof to justify the application of gene editing technology to enhance human traits. It is worth noting that this procedural requirement also complies with the allocation of responsibilities in *the Measures*.

Third, the precautionary principle mandates that anyone potentially impacted by HGE participate in the decision-making process. This necessitates a transparent decision-making process. In this respect, by applying the precautionary principle early on, entities such as universities, scientific research institutions, and medical and health institutions ought to disclose safety protocols they follow and suggestions for regulating gene editing technology. Further, multi-stakeholders should enter into a dialogue, which may lead to the discussion of a multi-stakeholder governance model in the next subpoint.

### Develop a multi-stakeholder governance model

The ethical evaluation of HGE requires the involvement of at least three kinds of stakeholders: experts, the general public, and governments. Experts shall comprise geneticists, bioethicists, specialists in clinical medicine, and other professionals. They are obligated to deliver reports on ethical standards, technical feasibility, and risk assessment, including but not limited to off-target effects and intergenerational genetic risks. The general public, as the object of HGE and the final recipients of its benefits and risks, should include patients, NGO representatives, ordinary citizens, and opinion leaders on social media. The government, as guardians of the public interest, ought to be established as a cross-departmental collaborative institution by the Ministry of Science and Technology, the Ministry of Justice, the National Health Commission, and other relevant authorities.

At present, stakeholder communication persists as the conventional “top-down” model. In March 2019, the Ministry of Science and Technology released the Regulations on the Safety Management of Biotechnology Research and Development (Draft for Comments) (the *Regulations Draft*), suggesting a model for experts, the government, and the public to communicate about the ethical governance of gene editing technology in China. Article 4, Paragraph 1 of the *Regulations Draft* unequivocally guarantees the freedom and development of research while safeguarding lawful rights and interests. To attain this objective, paragraph 2 of this Article mandates that the public must comprehend science and its associated risks through improved publicity and education. It appears that the government and experts serve as educators of technical risks, while the public functions as the learner; the former disseminate risk information to the latter in a “top-down” model, persuading the public to comprehend and accept technological risks. This model is highly efficient; yet, there is a lack of effective public engagement.

This paper proposes to establish a multi-stakeholder collaborative governance model to facilitate effective communication among stakeholders. Firstly, we suggest creating a public information platform. This platform will provide: (1) risk warning information, including real-time updates on worldwide and Chinese clinical trials and incidents associated with HGE; (2) updates on the application, rejection, and approval projects of HGE, along with real-time progress reports on ongoing HGE research and development initiatives in various contexts; (3) public involvement portal, encompassing the establishment of an online interface for opinion submission, delineation of response deadlines, engagement in voting for research and development project approvals, and implementation of follow-up mechanisms; (4) expert Q&A System, which can consistently disseminate public inquiries and expert replies; (5) government response system that offer hotlines or platform ports to address public inquiries, concerns, and regulatory recommendations pertaining to HGE. Secondly, during the pre-review phase of HGE application, a pre-review committee can be formed to ensure a substantial public participation ratio to address the primary risk concerns of the public. Finally, during the dynamic monitoring phase of HGE, a circuit breaker mechanism can be instituted. If public dissent on a specific HGE is above a designated level, the process will be halted and reevaluated until a new consensus is achieved.

The multi-stakeholder collaborative governance model is able to rectify the expert-driven model’s disregard for the general public’s knowledge, experience, and acceptance regarding HGE. Notably, stakeholder dialogue can improve the efficacy of the precautionary principle in mitigating social risks. To elucidate the relationship between the two, consider a research institution planning to undertake a clinical trial of a gene-editing intervention aimed at enhancing immunity in newborns against diabetes, a highly prevalent disease in China. To effectively initiate the project, the research institution must reveal and demonstrate during the application phase that the project remains beneath the safety threshold for individual health and societal risks. In the consensus-building phase, a pre-review committee, consisting of representatives from government departments, research institutions, and the public, is assembled. Following expert elucidations and the addressing of public apprehensions, debates, and hearings, a vote can be commenced. To contest the safety threshold set by the implementation of the precautionary principle, a supermajority such as three-quarters of the votes is necessary. In this way, stakeholders are substantially involved, but the precautionary principle remains the benchmark in the ethical evaluation of HGE.

### Establish regional ethics review centers

Given the possibility of conflicts of interest between research, applications, and the society at large, it seems imperative to establish an independent ethical review process that is institutionalized. China currently has a three-tiered system of ethical review committees: the national, provincial, and internal committees of medical institutes. In biomedical research involving humans, ethical review committees at all levels are crucial, yet there are two problems.

The first problem lies in their lack of independence. For instance, it is difficult to guarantee the independence of the internal ethics review committee, because its day-to-day operations are dependent on medical institutions. In response to this situation, as proposed by the *Regulation*, Sichuan Province, Shandong Province, Shanghai, Guangdong Province, Beijing, and other places have investigated the establishment of regional ethics review centers. With greater independence, the regional ethics review centers can conduct ethical reviews of pertinent institutions throughout the entire region. It is no longer dependent on funding from medical and research institutions.

The unifying standard of such ethics review committees and centers is the focus of the second problem. We suggest that this problem could be addressed by creating a central online registry that would record the ethics review outcomes and allow them to be searched and referred to easily. This registry is analogous to China Judgements Online, where judicial decisions are published to ensure consistent judgments in similar cases. In addition, there could be a section where members of ethics review committees and centers can discuss typical cases to promote the unification of ethical review standards.

## Conclusion

This paper systematically proposes the methods for promoting ethics for HGE in China. Internationally, ethical guidelines on HGE has gradually developed and the typological approach by defining the differences between enhancement and therapy has been abandoned. Domestically, on the one hand, both Chinese academics and the public have an open attitude toward HGE; on the other hand, certain industry calls for broad application of gene editing technology. Against this backdrop, this paper puts forth an ethical framework centered on three distinct but connected issues. First, the precautionary principle should be applied as an overarching benchmark. Second, a multi-stakeholder collaborative governance model including a system of coordinated communication between different stakeholders should be created. Lastly, regional ethics review centers and a central online registry should be established to improve the quality of ethical review.

The proposed framework requires a gradual and flexible process in which consensus can be gradually built up. For example, creating regional ethics review centers requires more than just a top-level design; it also requires ongoing exploration and improvement, which is equivalent to feeling the stones as you cross the river. Overall, we believe that China will establish a scientific and complete ethical framework for HGE at an appropriate time.

## Data Availability

The raw data supporting the conclusion of this article will be made available by the authors, without undue reservation.
